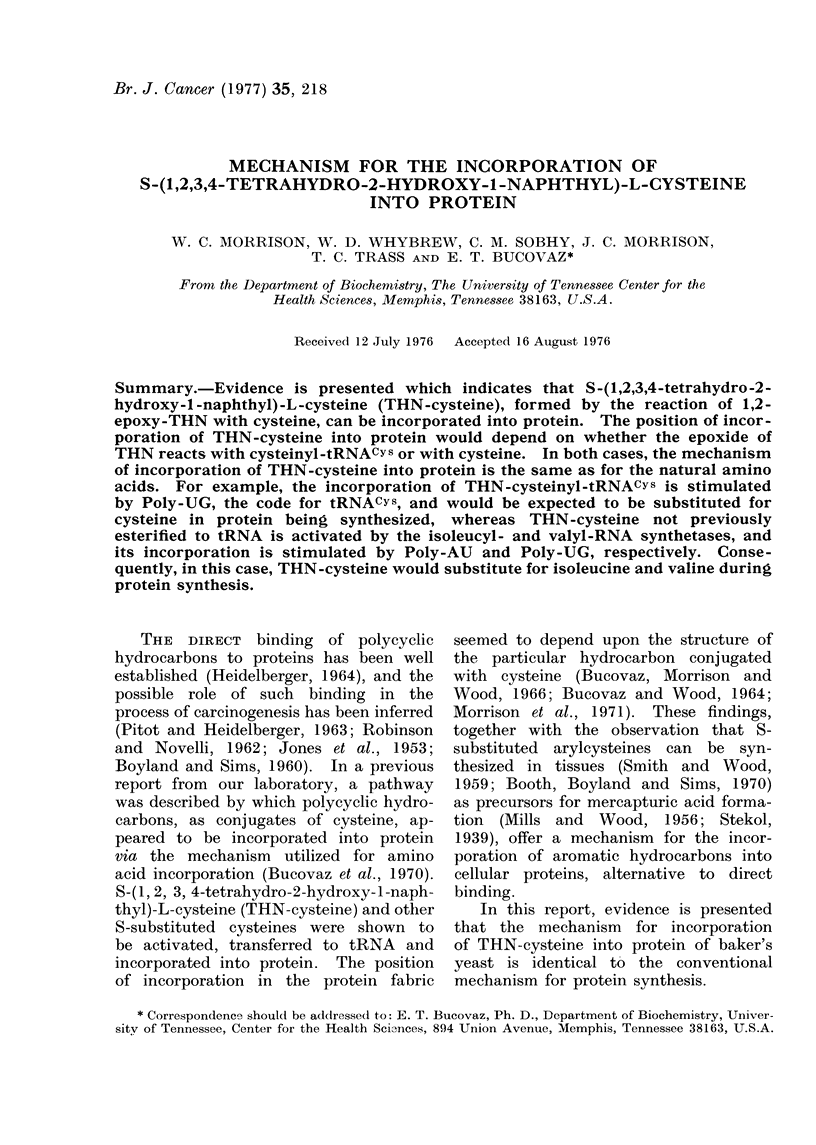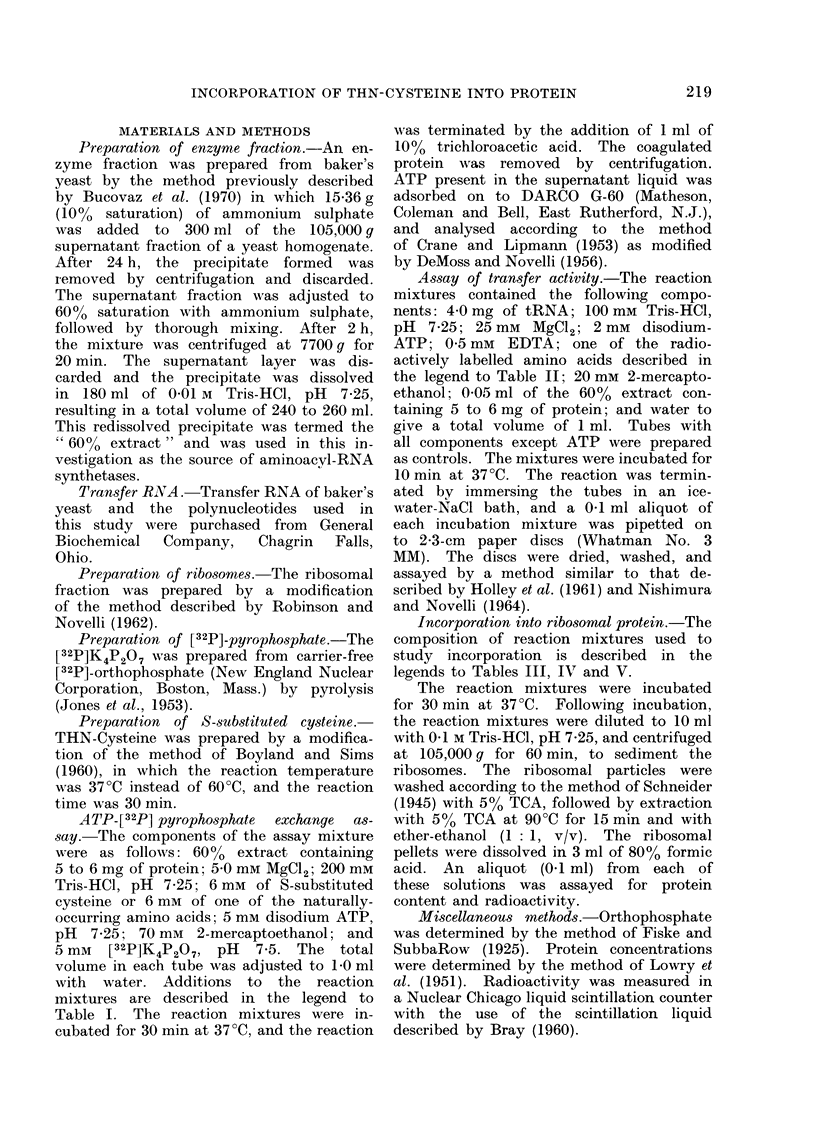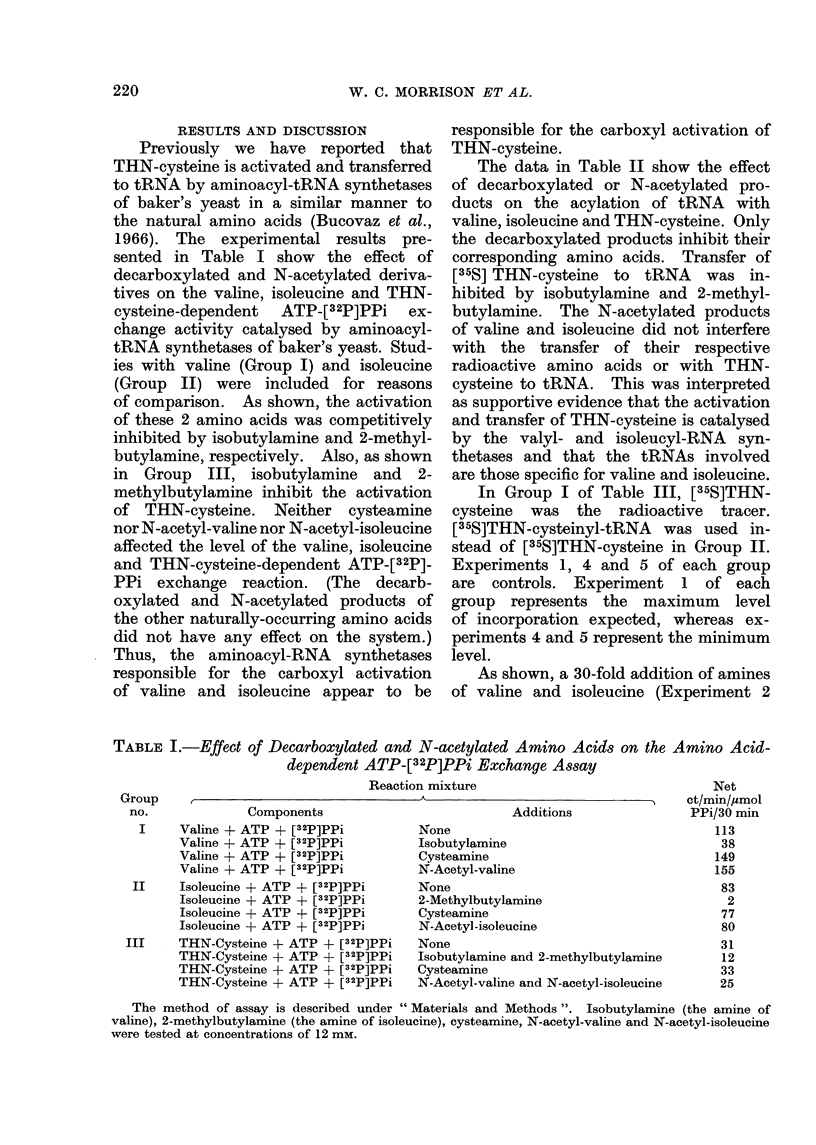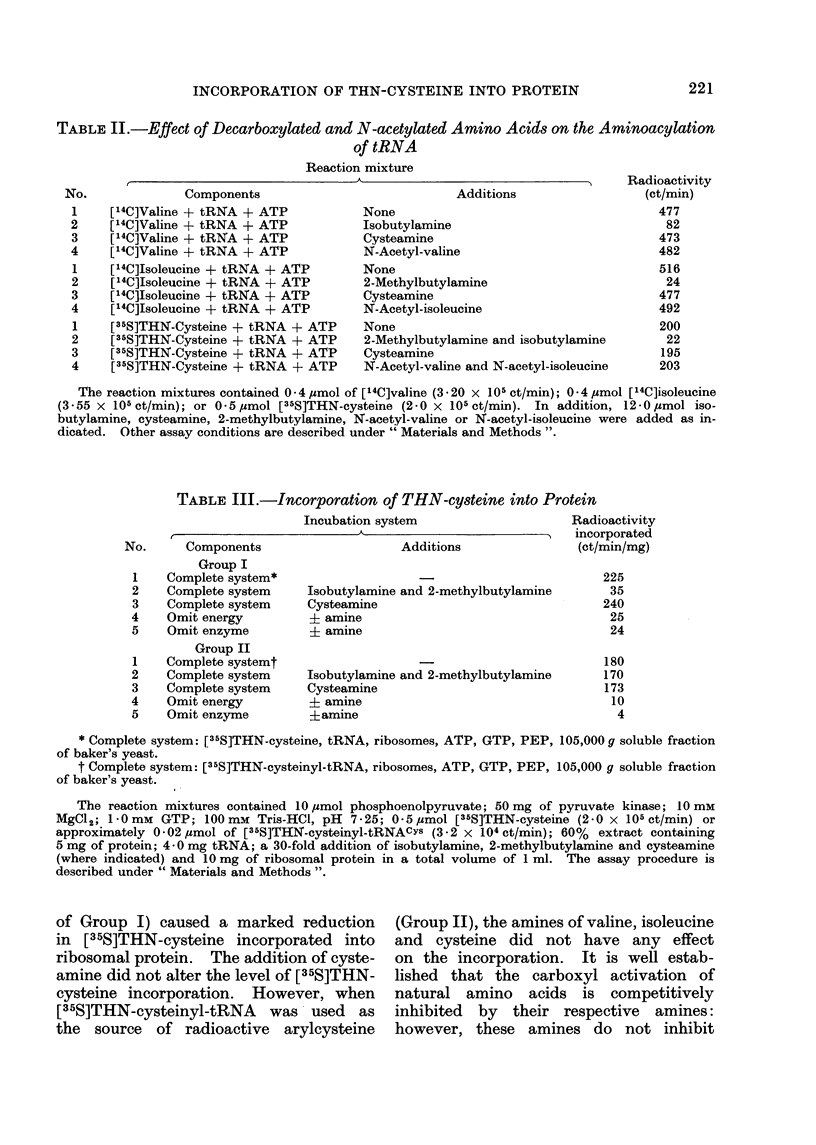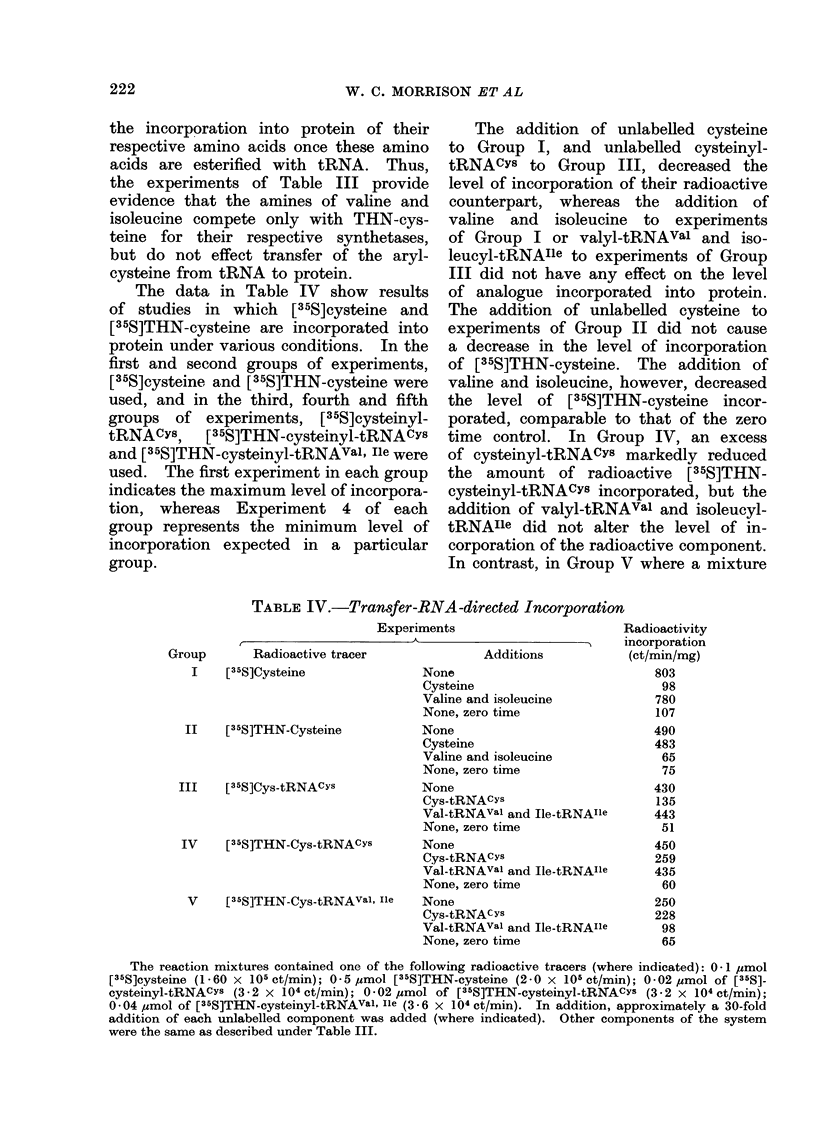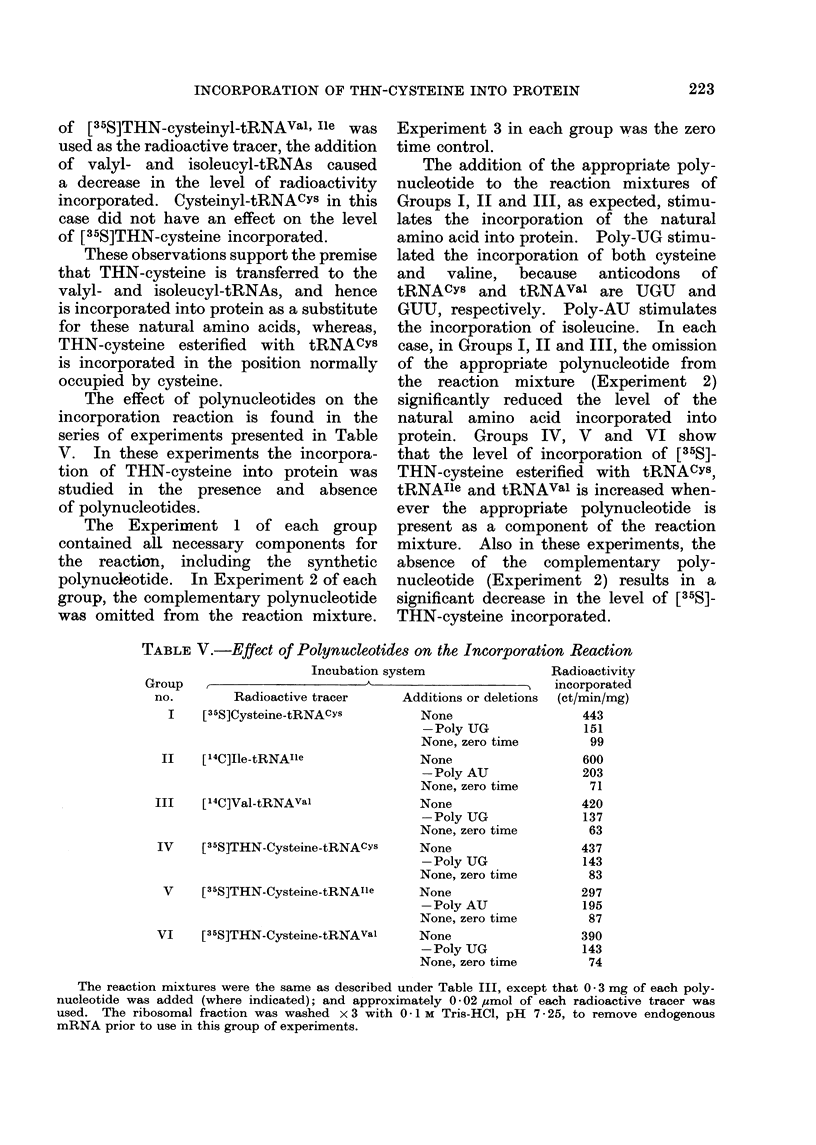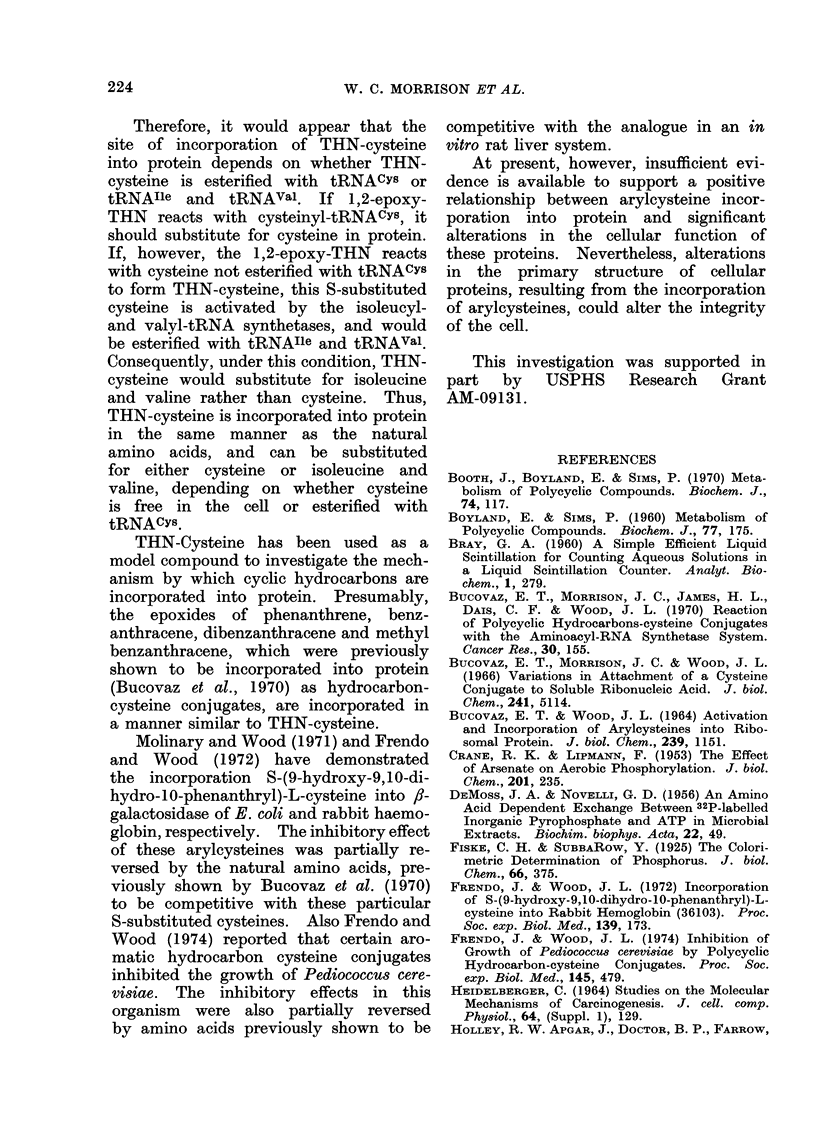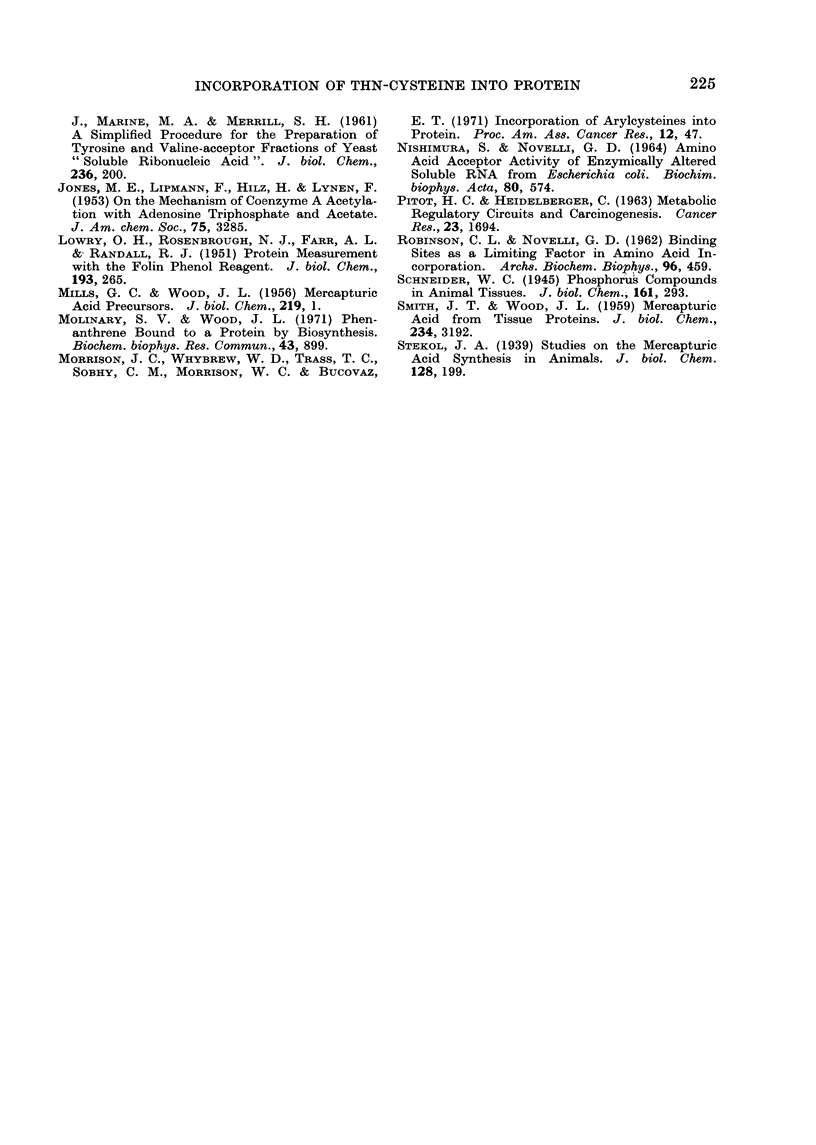# Mechanism for the incorporation of S-(1,2,3,4-tetrahydro-2-hydroxy-1-naphthyl)-L-cysteine into protein.

**DOI:** 10.1038/bjc.1977.29

**Published:** 1977-02

**Authors:** W. C. Morrison, W. D. Whybrew, C. M. Sobhy, J. C. Morrison, T. C. Trass, E. T. Bucovaz

## Abstract

Evidence is presented which indicates that S-(1,2,3,4-tetrahydro-2-hydroxy-1-naphthyl)-L-cysteine (THN-cysteine), formed by the reaction of 1,2-epoxy-THN with cysteine, can be incorporated into protein; The position of incorporation of THN-cysteine into protein would depend on whether the epoxide of THN reacts with cysteinyl-tRNACyS or with cysteine. In both cases, the mechanism of incorporation of THN-cysteine into protein is the same as for the natural amino acids. For example, the incorporation of THN-cysteinyl-tRNACyS is stimulated by Poly-UG, the code for tRNACyS, and would be expected to be substituted for cysteine in protein being synthesized, whereas THN-cysteine not previously esterified to tRNA is activated by the isoleucyl- and valyl-RNA synthetases, and its incorporation is stimulated by Poly-AU and Poly-UG, respectively. Consequently, in this case, THN-cysteine would substitute for isoleucine and valine during protein synthesis.


*Br. J. Cancer* (1977) **35**, 218

### MECHANISM FOR THE INCORPORATION OF S-(1,2,3,4-TETRAHYDRO-2-HYDROXY-1-NAPHTHYL)-L-CYSTEINE INTO PROTEIN

W. C. MORRISON, W. D. WHYBREW, C. M. SOBHY, J. C. MORRISON, T. C. TRASS AND E. T. BUCOVAZ*

*From the Department of Biochemistry, The University of Tennessee Center for the Health Sciences, Memphis, Tennessee* 38163, *U.S.A.*

Received 12 July 1976 Accepted 16 August 1976

**Summary.—Evidence is presented which indicates that S-(1,2,3,4-tetrahydro-2-hydroxy-1-naphthyl)-L-cysteine (THN-cysteine), formed by the reaction of 1,2-epoxy-THN with cysteine, can be incorporated into protein. The position of incorporation of THN-cysteine into protein would depend on whether the epoxide of THN reacts with cysteinyl-tRNA$^{Cys}$ or with cysteine. In both cases, the mechanism of incorporation of THN-cysteine into protein is the same as for the natural amino acids. For example, the incorporation of THN-cysteinyl-tRNA$^{Cys}$ is stimulated by Poly-UG, the code for tRNA$^{Cys}$, and would be expected to be substituted for cysteine in protein being synthesized, whereas THN-cysteine not previously esterified to tRNA is activated by the isoleucyl- and valyl-RNA synthetases, and its incorporation is stimulated by Poly-AU and Poly-UG, respectively. Consequently, in this case, THN-cysteine would substitute for isoleucine and valine during protein synthesis.**

THE DIRECT binding of polycyclic hydrocarbons to proteins has been well established (Heidelberger, 1964), and the possible role of such binding in the process of carcinogenesis has been inferred (Pitot and Heidelberger, 1963; Robinson and Novelli, 1962; Jones *et al.*, 1953; Boyland and Sims, 1960). In a previous report from our laboratory, a pathway was described by which polycyclic hydrocarbons, as conjugates of cysteine, appeared to be incorporated into protein *via* the mechanism utilized for amino acid incorporation (Bucovaz *et al.*, 1970). S-(1, 2, 3, 4-tetrahydro-2-hydroxy-1-naphthyl)-L-cysteine (THN-cysteine) and other S-substituted cysteines were shown to be activated, transferred to tRNA and incorporated into protein. The position of incorporation in the protein fabric seemed to depend upon the structure of the particular hydrocarbon conjugated with cysteine (Bucovaz, Morrison and Wood, 1966; Bucovaz and Wood, 1964; Morrison *et al.*, 1971). These findings, together with the observation that S-substituted arylcysteines can be synthesized in tissues (Smith and Wood, 1959; Booth, Boyland and Sims, 1970) as precursors for mercapturic acid formation (Mills and Wood, 1956; Stekol, 1939), offer a mechanism for the incorporation of aromatic hydrocarbons into cellular proteins, alternative to direct binding.

In this report, evidence is presented that the mechanism for incorporation of THN-cysteine into protein of baker's yeast is identical to the conventional mechanism for protein synthesis.

* Correspondence should be addressed to: E. T. Bucovaz, Ph. D., Department of Biochemistry, University of Tennessee, Center for the Health Sciences, 894 Union Avenue, Memphis, Tennessee 38163, U.S.A.

### MATERIALS AND METHODS

*Preparation of enzyme fraction.*—An enzyme fraction was prepared from baker's yeast by the method previously described by Bucovaz *et al.* (1970) in which 15·36 g (10% saturation) of ammonium sulphate was added to 300 ml of the 105,000 *g* supernatant fraction of a yeast homogenate. After 24 h, the precipitate formed was removed by centrifugation and discarded. The supernatant fraction was adjusted to 60% saturation with ammonium sulphate, followed by thorough mixing. After 2 h, the mixture was centrifuged at 7700 *g* for 20 min. The supernatant layer was discarded and the precipitate was dissolved in 180 ml of 0·01 M Tris-HCl, pH 7·25, resulting in a total volume of 240 to 260 ml. This redissolved precipitate was termed the "60% extract" and was used in this investigation as the source of aminoacyl-RNA synthetases.

*Transfer RNA.*—Transfer RNA of baker's yeast and the polynucleotides used in this study were purchased from General Biochemical Company, Chagrin Falls, Ohio.

*Preparation of ribosomes.*—The ribosomal fraction was prepared by a modification of the method described by Robinson and Novelli (1962).

*Preparation of* [$^{32}$P]-*pyrophosphate.*—The [$^{32}$P]$K_4P_2O_7$ was prepared from carrier-free [$^{32}$P]-orthophosphate (New England Nuclear Corporation, Boston, Mass.) by pyrolysis (Jones *et al.*, 1953).

*Preparation of S-substituted cysteine.*—THN-Cysteine was prepared by a modification of the method of Boyland and Sims (1960), in which the reaction temperature was 37°C instead of 60°C, and the reaction time was 30 min.

*ATP-*[$^{32}$*P*] *pyrophosphate exchange assay.*—The components of the assay mixture were as follows: 60% extract containing 5 to 6 mg of protein; 5·0 mM $MgCl_2$; 200 mM Tris-HCl, pH 7·25; 6 mM of S-substituted cysteine or 6 mM of one of the naturally-occurring amino acids; 5 mM disodium ATP, pH 7·25; 70 mM 2-mercaptoethanol; and 5 mM [$^{32}$P]$K_4P_2O_7$, pH 7·5. The total volume in each tube was adjusted to 1·0 ml with water. Additions to the reaction mixtures are described in the legend to Table I. The reaction mixtures were incubated for 30 min at 37°C, and the reaction was terminated by the addition of 1 ml of 10% trichloroacetic acid. The coagulated protein was removed by centrifugation. ATP present in the supernatant liquid was adsorbed on to DARCO G-60 (Matheson, Coleman and Bell, East Rutherford, N.J.), and analysed according to the method of Crane and Lipmann (1953) as modified by DeMoss and Novelli (1956).

*Assay of transfer activity.*—The reaction mixtures contained the following components: 4·0 mg of tRNA; 100 mM Tris-HCl, pH 7·25; 25 mM $MgCl_2$; 2 mM disodium-ATP; 0·5 mM EDTA; one of the radioactively labelled amino acids described in the legend to Table II; 20 mM 2-mercaptoethanol; 0·05 ml of the 60% extract containing 5 to 6 mg of protein; and water to give a total volume of 1 ml. Tubes with all components except ATP were prepared as controls. The mixtures were incubated for 10 min at 37°C. The reaction was terminated by immersing the tubes in an ice-water-NaCl bath, and a 0·1 ml aliquot of each incubation mixture was pipetted on to 2·3-cm paper discs (Whatman No. 3 MM). The discs were dried, washed, and assayed by a method similar to that described by Holley *et al.* (1961) and Nishimura and Novelli (1964).

*Incorporation into ribosomal protein.*—The composition of reaction mixtures used to study incorporation is described in the legends to Tables III, IV and V.

The reaction mixtures were incubated for 30 min at 37°C. Following incubation, the reaction mixtures were diluted to 10 ml with 0·1 M Tris-HCl, pH 7·25, and centrifuged at 105,000 *g* for 60 min, to sediment the ribosomes. The ribosomal particles were washed according to the method of Schneider (1945) with 5% TCA, followed by extraction with 5% TCA at 90°C for 15 min and with ether-ethanol (1 : 1, v/v). The ribosomal pellets were dissolved in 3 ml of 80% formic acid. An aliquot (0·1 ml) from each of these solutions was assayed for protein content and radioactivity.

*Miscellaneous methods.*—Orthophosphate was determined by the method of Fiske and SubbaRow (1925). Protein concentrations were determined by the method of Lowry *et al.* (1951). Radioactivity was measured in a Nuclear Chicago liquid scintillation counter with the use of the scintillation liquid described by Bray (1960).

RESULTS AND DISCUSSION

Previously we have reported that THN-cysteine is activated and transferred to tRNA by aminoacyl-tRNA synthetases of baker's yeast in a similar manner to the natural amino acids (Bucovaz *et al.*, 1966). The experimental results presented in Table I show the effect of decarboxylated and N-acetylated derivatives on the valine, isoleucine and THN-cysteine-dependent ATP-[$^{32}$P]PPi exchange activity catalysed by aminoacyl-tRNA synthetases of baker's yeast. Studies with valine (Group I) and isoleucine (Group II) were included for reasons of comparison. As shown, the activation of these 2 amino acids was competitively inhibited by isobutylamine and 2-methylbutylamine, respectively. Also, as shown in Group III, isobutylamine and 2-methylbutylamine inhibit the activation of THN-cysteine. Neither cysteamine nor N-acetyl-valine nor N-acetyl-isoleucine affected the level of the valine, isoleucine and THN-cysteine-dependent ATP-[$^{32}$P]-PPi exchange reaction. (The decarboxylated and N-acetylated products of the other naturally-occurring amino acids did not have any effect on the system.) Thus, the aminoacyl-RNA synthetases responsible for the carboxyl activation of valine and isoleucine appear to be responsible for the carboxyl activation of THN-cysteine.

The data in Table II show the effect of decarboxylated or N-acetylated products on the acylation of tRNA with valine, isoleucine and THN-cysteine. Only the decarboxylated products inhibit their corresponding amino acids. Transfer of [$^{35}$S] THN-cysteine to tRNA was inhibited by isobutylamine and 2-methylbutylamine. The N-acetylated products of valine and isoleucine did not interfere with the transfer of their respective radioactive amino acids or with THN-cysteine to tRNA. This was interpreted as supportive evidence that the activation and transfer of THN-cysteine is catalysed by the valyl- and isoleucyl-RNA synthetases and that the tRNAs involved are those specific for valine and isoleucine.

In Group I of Table III, [$^{35}$S]THN-cysteine was the radioactive tracer. [$^{35}$S]THN-cysteinyl-tRNA was used instead of [$^{35}$S]THN-cysteine in Group II. Experiments 1, 4 and 5 of each group are controls. Experiment 1 of each group represents the maximum level of incorporation expected, whereas experiments 4 and 5 represent the minimum level.

As shown, a 30-fold addition of amines of valine and isoleucine (Experiment 2

TABLE I.—*Effect of Decarboxylated and N-acetylated Amino Acids on the Amino Acid-dependent ATP-[$^{32}$P]PPi Exchange Assay*

| Group no. | Reaction mixture: Components | Additions | Net ct/min/μmol PPi/30 min |
|---|---|---|---|
| I | Valine + ATP + [$^{32}$P]PPi | None | 113 |
| | Valine + ATP + [$^{32}$P]PPi | Isobutylamine | 38 |
| | Valine + ATP + [$^{32}$P]PPi | Cysteamine | 149 |
| | Valine + ATP + [$^{32}$P]PPi | N-Acetyl-valine | 155 |
| II | Isoleucine + ATP + [$^{32}$P]PPi | None | 83 |
| | Isoleucine + ATP + [$^{32}$P]PPi | 2-Methylbutylamine | 2 |
| | Isoleucine + ATP + [$^{32}$P]PPi | Cysteamine | 77 |
| | Isoleucine + ATP + [$^{32}$P]PPi | N-Acetyl-isoleucine | 80 |
| III | THN-Cysteine + ATP + [$^{32}$P]PPi | None | 31 |
| | THN-Cysteine + ATP + [$^{32}$P]PPi | Isobutylamine and 2-methylbutylamine | 12 |
| | THN-Cysteine + ATP + [$^{32}$P]PPi | Cysteamine | 33 |
| | THN-Cysteine + ATP + [$^{32}$P]PPi | N-Acetyl-valine and N-acetyl-isoleucine | 25 |

The method of assay is described under " Materials and Methods ". Isobutylamine (the amine of valine), 2-methylbutylamine (the amine of isoleucine), cysteamine, N-acetyl-valine and N-acetyl-isoleucine were tested at concentrations of 12 mM.

TABLE II.—*Effect of Decarboxylated and N-acetylated Amino Acids on the Aminoacylation of tRNA*

| No. | Reaction mixture: Components | Reaction mixture: Additions | Radioactivity (ct/min) |
|---|---|---|---|
| 1 | [$^{14}$C]Valine + tRNA + ATP | None | 477 |
| 2 | [$^{14}$C]Valine + tRNA + ATP | Isobutylamine | 82 |
| 3 | [$^{14}$C]Valine + tRNA + ATP | Cysteamine | 473 |
| 4 | [$^{14}$C]Valine + tRNA + ATP | N-Acetyl-valine | 482 |
| 1 | [$^{14}$C]Isoleucine + tRNA + ATP | None | 516 |
| 2 | [$^{14}$C]Isoleucine + tRNA + ATP | 2-Methylbutylamine | 24 |
| 3 | [$^{14}$C]Isoleucine + tRNA + ATP | Cysteamine | 477 |
| 4 | [$^{14}$C]Isoleucine + tRNA + ATP | N-Acetyl-isoleucine | 492 |
| 1 | [$^{35}$S]THN-Cysteine + tRNA + ATP | None | 200 |
| 2 | [$^{35}$S]THN-Cysteine + tRNA + ATP | 2-Methylbutylamine and isobutylamine | 22 |
| 3 | [$^{35}$S]THN-Cysteine + tRNA + ATP | Cysteamine | 195 |
| 4 | [$^{35}$S]THN-Cysteine + tRNA + ATP | N-Acetyl-valine and N-acetyl-isoleucine | 203 |

The reaction mixtures contained 0·4 μmol of [$^{14}$C]valine (3·20 × $10^5$ ct/min); 0·4 μmol [$^{14}$C]isoleucine (3·55 × $10^5$ ct/min); or 0·5 μmol [$^{35}$S]THN-cysteine (2·0 × $10^5$ ct/min). In addition, 12·0 μmol isobutylamine, cysteamine, 2-methylbutylamine, N-acetyl-valine or N-acetyl-isoleucine were added as indicated. Other assay conditions are described under " Materials and Methods ".

TABLE III.—*Incorporation of THN-cysteine into Protein*

| No. | Incubation system: Components | Incubation system: Additions | Radioactivity incorporated (ct/min/mg) |
|---|---|---|---|
| | Group I | | |
| 1 | Complete system* | — | 225 |
| 2 | Complete system | Isobutylamine and 2-methylbutylamine | 35 |
| 3 | Complete system | Cysteamine | 240 |
| 4 | Omit energy | ± amine | 25 |
| 5 | Omit enzyme | ± amine | 24 |
| | Group II | | |
| 1 | Complete system† | — | 180 |
| 2 | Complete system | Isobutylamine and 2-methylbutylamine | 170 |
| 3 | Complete system | Cysteamine | 173 |
| 4 | Omit energy | ± amine | 10 |
| 5 | Omit enzyme | ±amine | 4 |

* Complete system: [$^{35}$S]THN-cysteine, tRNA, ribosomes, ATP, GTP, PEP, 105,000 *g* soluble fraction of baker's yeast.

† Complete system: [$^{35}$S]THN-cysteinyl-tRNA, ribosomes, ATP, GTP, PEP, 105,000 *g* soluble fraction of baker's yeast.

The reaction mixtures contained 10 μmol phosphoenolpyruvate; 50 mg of pyruvate kinase; 10 mM $MgCl_2$; 1·0 mM GTP; 100 mM Tris-HCl, pH 7·25; 0·5 μmol [$^{35}$S]THN-cysteine (2·0 × $10^5$ ct/min) or approximately 0·02 μmol of [$^{35}$S]THN-cysteinyl-$tRNA^{Cys}$ (3·2 × $10^4$ ct/min); 60% extract containing 5 mg of protein; 4·0 mg tRNA; a 30-fold addition of isobutylamine, 2-methylbutylamine and cysteamine (where indicated) and 10 mg of ribosomal protein in a total volume of 1 ml. The assay procedure is described under " Materials and Methods ".

of Group I) caused a marked reduction in [$^{35}$S]THN-cysteine incorporated into ribosomal protein. The addition of cysteamine did not alter the level of [$^{35}$S]THN-cysteine incorporation. However, when [$^{35}$S]THN-cysteinyl-tRNA was used as the source of radioactive arylcysteine (Group II), the amines of valine, isoleucine and cysteine did not have any effect on the incorporation. It is well established that the carboxyl activation of natural amino acids is competitively inhibited by their respective amines: however, these amines do not inhibit the incorporation into protein of their respective amino acids once these amino acids are esterified with tRNA. Thus, the experiments of Table III provide evidence that the amines of valine and isoleucine compete only with THN-cysteine for their respective synthetases, but do not effect transfer of the arylcysteine from tRNA to protein.

The data in Table IV show results of studies in which [$^{35}$S]cysteine and [$^{35}$S]THN-cysteine are incorporated into protein under various conditions. In the first and second groups of experiments, [$^{35}$S]cysteine and [$^{35}$S]THN-cysteine were used, and in the third, fourth and fifth groups of experiments, [$^{35}$S]cysteinyl-tRNA$^{Cys}$, [$^{35}$S]THN-cysteinyl-tRNA$^{Cys}$ and [$^{35}$S]THN-cysteinyl-tRNA$^{Val, Ile}$ were used. The first experiment in each group indicates the maximum level of incorporation, whereas Experiment 4 of each group represents the minimum level of incorporation expected in a particular group.

The addition of unlabelled cysteine to Group I, and unlabelled cysteinyl-tRNA$^{Cys}$ to Group III, decreased the level of incorporation of their radioactive counterpart, whereas the addition of valine and isoleucine to experiments of Group I or valyl-tRNA$^{Val}$ and isoleucyl-tRNA$^{Ile}$ to experiments of Group III did not have any effect on the level of analogue incorporated into protein. The addition of unlabelled cysteine to experiments of Group II did not cause a decrease in the level of incorporation of [$^{35}$S]THN-cysteine. The addition of valine and isoleucine, however, decreased the level of [$^{35}$S]THN-cysteine incorporated, comparable to that of the zero time control. In Group IV, an excess of cysteinyl-tRNA$^{Cys}$ markedly reduced the amount of radioactive [$^{35}$S]THN-cysteinyl-tRNA$^{Cys}$ incorporated, but the addition of valyl-tRNA$^{Val}$ and isoleucyl-tRNA$^{Ile}$ did not alter the level of incorporation of the radioactive component. In contrast, in Group V where a mixture

TABLE IV.—*Transfer-RNA-directed Incorporation*

| | Experiments | | Radioactivity incorporation |
|---|---|---|---|
| Group | Radioactive tracer | Additions | (ct/min/mg) |
| I | [$^{35}$S]Cysteine | None | 803 |
| | | Cysteine | 98 |
| | | Valine and isoleucine | 780 |
| | | None, zero time | 107 |
| II | [$^{35}$S]THN-Cysteine | None | 490 |
| | | Cysteine | 483 |
| | | Valine and isoleucine | 65 |
| | | None, zero time | 75 |
| III | [$^{35}$S]Cys-tRNA$^{Cys}$ | None | 430 |
| | | Cys-tRNA$^{Cys}$ | 135 |
| | | Val-tRNA$^{Val}$ and Ile-tRNA$^{Ile}$ | 443 |
| | | None, zero time | 51 |
| IV | [$^{35}$S]THN-Cys-tRNA$^{Cys}$ | None | 450 |
| | | Cys-tRNA$^{Cys}$ | 259 |
| | | Val-tRNA$^{Val}$ and Ile-tRNA$^{Ile}$ | 435 |
| | | None, zero time | 60 |
| V | [$^{35}$S]THN-Cys-tRNA$^{Val, Ile}$ | None | 250 |
| | | Cys-tRNA$^{Cys}$ | 228 |
| | | Val-tRNA$^{Val}$ and Ile-tRNA$^{Ile}$ | 98 |
| | | None, zero time | 65 |

The reaction mixtures contained one of the following radioactive tracers (where indicated): 0·1 $\mu$mol [$^{35}$S]cysteine ($1 \cdot 60 \times 10^5$ ct/min); 0·5 $\mu$mol [$^{35}$S]THN-cysteine ($2 \cdot 0 \times 10^5$ ct/min); 0·02 $\mu$mol of [$^{35}$S]-cysteinyl-tRNA$^{Cys}$ ($3 \cdot 2 \times 10^4$ ct/min); 0·02 $\mu$mol of [$^{35}$S]THN-cysteinyl-tRNA$^{Cys}$ ($3 \cdot 2 \times 10^4$ ct/min); 0·04 $\mu$mol of [$^{35}$S]THN-cysteinyl-tRNA$^{Val, Ile}$ ($3 \cdot 6 \times 10^4$ ct/min). In addition, approximately a 30-fold addition of each unlabelled component was added (where indicated). Other components of the system were the same as described under Table III.

of [$^{35}$S]THN-cysteinyl-tRNA$^{Val, Ile}$ was used as the radioactive tracer, the addition of valyl- and isoleucyl-tRNAs caused a decrease in the level of radioactivity incorporated. Cysteinyl-tRNA$^{Cys}$ in this case did not have an effect on the level of [$^{35}$S]THN-cysteine incorporated.

These observations support the premise that THN-cysteine is transferred to the valyl- and isoleucyl-tRNAs, and hence is incorporated into protein as a substitute for these natural amino acids, whereas, THN-cysteine esterified with tRNA$^{Cys}$ is incorporated in the position normally occupied by cysteine.

The effect of polynucleotides on the incorporation reaction is found in the series of experiments presented in Table V. In these experiments the incorporation of THN-cysteine into protein was studied in the presence and absence of polynucleotides.

The Experiment 1 of each group contained all necessary components for the reaction, including the synthetic polynucleotide. In Experiment 2 of each group, the complementary polynucleotide was omitted from the reaction mixture. Experiment 3 in each group was the zero time control.

The addition of the appropriate polynucleotide to the reaction mixtures of Groups I, II and III, as expected, stimulates the incorporation of the natural amino acid into protein. Poly-UG stimulated the incorporation of both cysteine and valine, because anticodons of tRNA$^{Cys}$ and tRNA$^{Val}$ are UGU and GUU, respectively. Poly-AU stimulates the incorporation of isoleucine. In each case, in Groups I, II and III, the omission of the appropriate polynucleotide from the reaction mixture (Experiment 2) significantly reduced the level of the natural amino acid incorporated into protein. Groups IV, V and VI show that the level of incorporation of [$^{35}$S]-THN-cysteine esterified with tRNA$^{Cys}$, tRNA$^{Ile}$ and tRNA$^{Val}$ is increased whenever the appropriate polynucleotide is present as a component of the reaction mixture. Also in these experiments, the absence of the complementary polynucleotide (Experiment 2) results in a significant decrease in the level of [$^{35}$S]-THN-cysteine incorporated.

TABLE V.—*Effect of Polynucleotides on the Incorporation Reaction*

| Group no. | Incubation system: Radioactive tracer | Incubation system: Additions or deletions | Radioactivity incorporated (ct/min/mg) |
|---|---|---|---|
| I | [$^{35}$S]Cysteine-tRNA$^{Cys}$ | None | 443 |
| | | −Poly UG | 151 |
| | | None, zero time | 99 |
| II | [$^{14}$C]Ile-tRNA$^{Ile}$ | None | 600 |
| | | −Poly AU | 203 |
| | | None, zero time | 71 |
| III | [$^{14}$C]Val-tRNA$^{Val}$ | None | 420 |
| | | −Poly UG | 137 |
| | | None, zero time | 63 |
| IV | [$^{35}$S]THN-Cysteine-tRNA$^{Cys}$ | None | 437 |
| | | −Poly UG | 143 |
| | | None, zero time | 83 |
| V | [$^{35}$S]THN-Cysteine-tRNA$^{Ile}$ | None | 297 |
| | | −Poly AU | 195 |
| | | None, zero time | 87 |
| VI | [$^{35}$S]THN-Cysteine-tRNA$^{Val}$ | None | 390 |
| | | −Poly UG | 143 |
| | | None, zero time | 74 |

The reaction mixtures were the same as described under Table III, except that 0·3 mg of each polynucleotide was added (where indicated); and approximately 0·02 $\mu$mol of each radioactive tracer was used. The ribosomal fraction was washed ×3 with 0·1 M Tris-HCl, pH 7·25, to remove endogenous mRNA prior to use in this group of experiments.

Therefore, it would appear that the site of incorporation of THN-cysteine into protein depends on whether THN-cysteine is esterified with $tRNA^{Cys}$ or $tRNA^{Ile}$ and $tRNA^{Val}$. If 1,2-epoxy-THN reacts with cysteinyl-$tRNA^{Cys}$, it should substitute for cysteine in protein. If, however, the 1,2-epoxy-THN reacts with cysteine not esterified with $tRNA^{Cys}$ to form THN-cysteine, this S-substituted cysteine is activated by the isoleucyl- and valyl-tRNA synthetases, and would be esterified with $tRNA^{Ile}$ and $tRNA^{Val}$. Consequently, under this condition, THN-cysteine would substitute for isoleucine and valine rather than cysteine. Thus, THN-cysteine is incorporated into protein in the same manner as the natural amino acids, and can be substituted for either cysteine or isoleucine and valine, depending on whether cysteine is free in the cell or esterified with $tRNA^{Cys}$.

THN-Cysteine has been used as a model compound to investigate the mechanism by which cyclic hydrocarbons are incorporated into protein. Presumably, the epoxides of phenanthrene, benzanthracene, dibenzanthracene and methyl benzanthracene, which were previously shown to be incorporated into protein (Bucovaz *et al.*, 1970) as hydrocarbon-cysteine conjugates, are incorporated in a manner similar to THN-cysteine.

Molinary and Wood (1971) and Frendo and Wood (1972) have demonstrated the incorporation S-(9-hydroxy-9,10-dihydro-10-phenanthryl)-L-cysteine into β-galactosidase of *E. coli* and rabbit haemoglobin, respectively. The inhibitory effect of these arylcysteines was partially reversed by the natural amino acids, previously shown by Bucovaz *et al.* (1970) to be competitive with these particular S-substituted cysteines. Also Frendo and Wood (1974) reported that certain aromatic hydrocarbon cysteine conjugates inhibited the growth of *Pediococcus cerevisiae*. The inhibitory effects in this organism were also partially reversed by amino acids previously shown to be competitive with the analogue in an *in vitro* rat liver system.

At present, however, insufficient evidence is available to support a positive relationship between arylcysteine incorporation into protein and significant alterations in the cellular function of these proteins. Nevertheless, alterations in the primary structure of cellular proteins, resulting from the incorporation of arylcysteines, could alter the integrity of the cell.

This investigation was supported in part by USPHS Research Grant AM-09131.

REFERENCES

Booth, J., Boyland, E. & Sims, P. (1970) Metabolism of Polycyclic Compounds. *Biochem. J.*, **74**, 117.

Boyland, E. & Sims, P. (1960) Metabolism of Polycyclic Compounds. *Biochem. J.*, **77**, 175.

Bray, G. A. (1960) A Simple Efficient Liquid Scintillation for Counting Aqueous Solutions in a Liquid Scintillation Counter. *Analyt. Biochem.*, **1**, 279.

Bucovaz, E. T., Morrison, J. C., James, H. L., Dais, C. F. & Wood, J. L. (1970) Reaction of Polycyclic Hydrocarbons-cysteine Conjugates with the Aminoacyl-RNA Synthetase System. *Cancer Res.*, **30**, 155.

Bucovaz, E. T., Morrison, J. C. & Wood, J. L. (1966) Variations in Attachment of a Cysteine Conjugate to Soluble Ribonucleic Acid. *J. biol. Chem.*, **241**, 5114.

Bucovaz, E. T. & Wood, J. L. (1964) Activation and Incorporation of Arylcysteines into Ribosomal Protein. *J. biol. Chem.*, **239**, 1151.

Crane, R. K. & Lipmann, F. (1953) The Effect of Arsenate on Aerobic Phosphorylation. *J. biol. Chem.*, **201**, 235.

DeMoss, J. A. & Novelli, G. D. (1956) An Amino Acid Dependent Exchange Between $^{32}P$-labelled Inorganic Pyrophosphate and ATP in Microbial Extracts. *Biochim. biophys. Acta*, **22**, 49.

Fiske, C. H. & SubbaRow, Y. (1925) The Colorimetric Determination of Phosphorus. *J. biol. Chem.*, **66**, 375.

Frendo, J. & Wood, J. L. (1972) Incorporation of S-(9-hydroxy-9,10-dihydro-10-phenanthryl)-L-cysteine into Rabbit Hemoglobin (36103). *Proc. Soc. exp. Biol. Med.*, **139**, 173.

Frendo, J. & Wood, J. L. (1974) Inhibition of Growth of *Pediococcus cerevisiae* by Polycyclic Hydrocarbon-cysteine Conjugates. *Proc. Soc. exp. Biol. Med.*, **145**, 479.

Heidelberger, C. (1964) Studies on the Molecular Mechanisms of Carcinogenesis. *J. cell. comp. Physiol.*, **64**, (Suppl. 1), 129.

Holley, R. W. Apgar, J., Doctor, B. P., Farrow,

J., Marine, M. A. & Merrill, S. H. (1961) A Simplified Procedure for the Preparation of Tyrosine and Valine-acceptor Fractions of Yeast "Soluble Ribonucleic Acid". *J. biol. Chem.*, **236**, 200.

Jones, M. E., Lipmann, F., Hilz, H. & Lynen, F. (1953) On the Mechanism of Coenzyme A Acetylation with Adenosine Triphosphate and Acetate. *J. Am. chem. Soc.*, **75**, 3285.

Lowry, O. H., Rosenbrough, N. J., Farr, A. L. & Randall, R. J. (1951) Protein Measurement with the Folin Phenol Reagent. *J. biol. Chem.*, **193**, 265.

Mills, G. C. & Wood, J. L. (1956) Mercapturic Acid Precursors. *J. biol. Chem.*, **219**, 1.

Molinary, S. V. & Wood, J. L. (1971) Phenanthrene Bound to a Protein by Biosynthesis. *Biochem. biophys. Res. Commun.*, **43**, 899.

Morrison, J. C., Whybrew, W. D., Trass, T. C., Sobhy, C. M., Morrison, W. C. & Bucovaz, E. T. (1971) Incorporation of Arylcysteines into Protein. *Proc. Am. Ass. Cancer Res.*, **12**, 47.

Nishimura, S. & Novelli, G. D. (1964) Amino Acid Acceptor Activity of Enzymically Altered Soluble RNA from *Escherichia coli*. *Biochim. biophys. Acta*, **80**, 574.

Pitot, H. C. & Heidelberger, C. (1963) Metabolic Regulatory Circuits and Carcinogenesis. *Cancer Res.*, **23**, 1694.

Robinson, C. L. & Novelli, G. D. (1962) Binding Sites as a Limiting Factor in Amino Acid Incorporation. *Archs. Biochem. Biophys.*, **96**, 459.

Schneider, W. C. (1945) Phosphorus Compounds in Animal Tissues. *J. biol. Chem.*, **161**, 293.

Smith, J. T. & Wood, J. L. (1959) Mercapturic Acid from Tissue Proteins. *J. biol. Chem.*, **234**, 3192.

Stekol, J. A. (1939) Studies on the Mercapturic Acid Synthesis in Animals. *J. biol. Chem.* **128**, 199.